# Evaluation of Online Risk Assessment To Identify Rabies Exposures Among Health Care Workers — Utah, 2019

**DOI:** 10.15585/mmwr.mm6929a3

**Published:** 2020-07-24

**Authors:** Erin R. Whitehouse, Dallin Peterson, Keegan McCaffrey, Amit Eichenbaum, Randon Gruninger, Kristin K. Dascomb, Cherie Frame, Ryan Wallace, Jesse Bonwitt

**Affiliations:** ^1^Epidemic Intelligence Service, CDC; ^2^Poxvirus and Rabies Branch, Division of High Consequence Pathogens and Pathology, National Center for Emerging and Zoonotic Infectious Diseases, CDC; ^3^Utah Department of Health, Salt Lake City; ^4^University of Georgia, Athens; ^5^Division of Clinical Epidemiology and Infectious Diseases, Intermountain Medical Center, Murray, Utah.

On November 7, 2018, the Utah Department of Health (UDOH) reported the first confirmed human rabies death in the state since 1944 (*1*). The case occurred in a person who had been treated over a period of 19 days at four health care facilities and an emergency medical transport service across three counties and two states. Human rabies is preventable through preexposure or postexposure vaccination but is invariably fatal upon symptom onset. Timely identification of persons who might have been exposed to rabies virus is therefore crucial to administer postexposure prophylaxis (PEP). Because of the large number of health care workers who had been involved in the patient’s care, a standardized online risk assessment survey was developed by UDOH based on Advisory Committee on Immunization Practices recommendations (*2*). This online tool was evaluated for accuracy, acceptability, and administrative obligation by reviewing the results from the tool and conducting focus group discussions and a follow-up survey. Among 90 health care workers initially identified by the online risk assessment as being potentially exposed to infectious material, 74 were classified as exposed. All 74 health care workers received PEP following consultation with occupational health staff members, indicating a positive predictive value of the assessment tool of 82%. In a follow-up survey, 42 (76%) of the 55 respondents reported that they were satisfied with the assessment process. In focus group discussions, participants suggested that the survey could be improved by providing additional information about rabies exposures because many of them were unfamiliar with human-to-human rabies transmission. This evaluation highlighted the importance of adopting clear communication strategies, demonstrated the benefits of using an online risk assessment during a mass rabies exposure, and provided specific feedback for CDC to improve resources available for states and health care facilities after mass rabies exposures. 

Human-to-human transmission of rabies virus has only been confirmed among organ and tissue transplant recipients; however, because rabies virus has been isolated from tears, saliva, and nervous tissues of rabies patients, the possibility cannot be excluded (*2*). Because of the rarity of rabies and initial nonspecific signs and symptoms, patients with rabies sometimes have prolonged interactions with health care workers before diagnosis, which can result in multiple instances of exposure to potentially infectious materials. In such events, thorough risk assessments for potential rabies virus exposure, usually conducted by public health practitioners, are necessary to determine the need for PEP. Innovative methods that efficiently assess exposure risk and appropriately recommend PEP could improve the efficiency of health systems.

Within 48 hours of the 2018 Utah rabies case diagnosis, UDOH activated an Incident Command System and distributed the online risk assessment tool to infection prevention teams at four health care facilities and an emergency medical transport service. The infection prevention teams worked with supervisors to identify health care workers who might have been exposed, e-mailed them the risk assessment, and monitored completion of the assessment over the next 3 weeks.

The risk assessment tool (Supplementary material; https://stacks.cdc.gov/view/cdc/90520) was developed using Research Electronic Data Capture (REDCap) (*3*). The survey included questions about direct contact with certain infectious materials (cerebrospinal fluid [CSF], nervous tissue, saliva, respiratory secretions, or tears), and contact of infectious materials with mucous membranes (eyes, nose, and mouth) or broken skin (e.g., abrasion or cuts). Health care workers were asked whether they were involved in endotracheal intubation, tracheal tube maintenance, or oral care, and whether they were wearing appropriate personal protective equipment (PPE) or had direct contact with infectious materials during the procedure. An automated risk algorithm embedded in the online assessment provided recommendation for PEP if respondents reported any direct mucous membrane or broken skin contact with infectious materials. Health care workers were referred to occupational health staff members for in-person assessments if the algorithm determined that PEP was recommended or if further assessment was indicated (i.e., if health care workers reported additional exposures or concerns). The outcome of the online risk assessment was analyzed to assess the types and frequencies of exposures and determine the positive predictive value of the risk algorithm.

To understand knowledge gaps about human rabies among health care workers and to evaluate the acceptability of the online risk assessment, UDOH and CDC conducted focus group discussions with employees and infection prevention teams from the health care systems where the patient was hospitalized. Based on the results obtained from the focus groups, UDOH and CDC developed an online satisfaction survey in REDCap, which was sent to health care workers who completed the online risk assessment. Respondents were asked to rank their familiarity with rabies, level of concern, and satisfaction with the risk assessment process using a Likert scale and open-ended answers. Descriptive statistics were calculated using STATA software (version 14.0; StataCorp). This investigation was determined by CDC to be public health surveillance.*

The online risk assessment was completed by 242 health care workers in four facilities and one emergency medical service. The algorithm initially recommended 80 health care workers for PEP and 10 for additional follow-up with occupational health staff members. Among these 90 persons for whom a potential exposure could not be ruled out, 74 were classified as having been exposed and received PEP following consultation with occupational health, indicating a positive predictive value of the assessment tool of 82%. No rabies deaths were reported among health care workers more than 12 months after the event.

Among all 242 respondents, 140 (58%) reported no exposures, 74 (31%) reported performing procedures that could have placed them at risk for an exposure (e.g., intubation, oral care, needlestick), and 28 (12%) reported having had direct contact with infectious material not involving a medical procedure (e.g., CSF, tears, neural tissue, saliva, or respiratory secretions) (Figure); some respondents had multiple exposures and other exposure types such as laboratory exposures or other concerns not addressed in the survey. Among the 74 health care workers who performed tracheal or oral care (including intubation), 67 (91%) reported not wearing PPE to cover their eyes, nose, and mouth. Of these, 25 (37%) reported direct contact with respiratory secretions.

**FIGURE:**
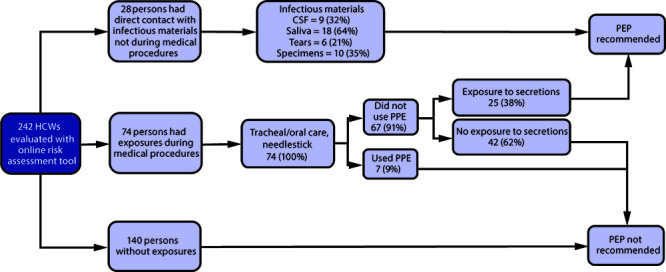
Health care worker exposures to potentially infectious materials* from a case of human rabies, by type of exposure, and postexposure prophylaxis recommendations based on an online risk assessment — Utah, 2019 **Abbreviations:** CSF = cerebrospinal fluid; HCWs = health care workers; PEP = postexposure prophylaxis rabies vaccination; PPE = personal protective equipment. * Laboratory specimen exposures included tears, respiratory secretions, saliva, CSF, and neural tissue; categories for the type of exposure were not mutually exclusive and do not show all possible exposure categories.

Among the 242 health care workers who completed the online risk assessment, 55 (23%) also responded to the follow-up satisfaction survey. Among those respondents, 35 (64%) indicated that they were not very familiar with rabies infection prevention or routes of exposure. Of the 55, 28 (51%) reported high levels of personal concern about exposures at the time of the patient’s rabies diagnosis. Unfamiliarity with rabies among some health care workers was also identified during focus group discussions. Health care workers reported being unfamiliar with clinical signs and transmission of human rabies and recommended use of PPE to prevent exposures.^†^ This resulted in anxiety among health care workers, illustrated by statements such as “I did not kiss my husband for 2 weeks” and “I slept on the sofa [out of fear of infecting my family].”

Health care workers reported initially receiving delayed and conflicting information about rabies transmission from their supervisors, the occupational health clinic, and Internet sources. Online resources about human-to-human transmission specific to hospital settings were reportedly difficult to find. Administrators explained that it took approximately 1 week to develop and distribute informational materials, a delay that exacerbated anxiety among health care workers.

Of the 55 respondents to the satisfaction survey, 42 (76%) were satisfied with the online risk assessment, and 48 (87%) recommended that it be used in future situations. Some reasons against using the risk assessment included unclear guidance concerning what constituted a rabies exposure, unclear and lengthy questions, concerns about the accuracy of the automated PEP algorithm, and insufficiently tailored questions for certain professions (e.g., laboratorians and housekeeping staff members). These concerns were also expressed during focus group discussions. Respondents suggested that the risk assessment should be used only as a screening tool, which would refer persons with elevated exposure risk to their health care providers for in-person assessments.

## Discussion

This evaluation found that the online risk assessment identified health care workers with potential exposures and was helpful and recommended by users for future use. However, the process could be improved by tailoring questions to specific audiences, clarifying exposure assessment questions, and including background information on rabies. Timely distribution of clear information in line with established risk communication principles could improve the process and alleviate health care worker anxiety (*4*). These findings suggest that an online risk assessment could be used to rapidly rule out nonexposures, while allowing thorough in-person assessment and counseling of potentially exposed persons.

In addition, this evaluation revealed suboptimal use of PPE among health care workers. Approximately 90% of health care workers who performed high-risk procedures reported not wearing adequate PPE while caring for a patient with encephalitis of unknown origin. Standard infection control precautions are sufficient to protect against most exposures to pathogens causing encephalitis (including rabies), and although the precautions are recommended while caring for all patients in a hospital setting, low adherence continues to be reported (*5*).

The findings in this report are subject to at least two limitations. First, because an additional qualitative risk assessment was performed by the occupational health clinic for workers who were considered exposed based on the online risk assessment result, it was not possible to ascertain whether the final PEP determination came from the online assessment. Second, the follow-up satisfaction survey was subject to recall and nonresponse bias because the survey was completed 5 months after the exposure window and only 55 of 242 health care workers responded.

Although rabies is rare in the United States, during the last 5 years, an average of 177 health care workers underwent an exposure risk assessment for every hospitalized human rabies patient (*6*–*9*) (Poxvirus and Rabies Branch, CDC, unpublished data). Because clinicians are recommended to consult with public health officials for nonroutine exposures, the workload placed on health departments by rabies exposures in health care settings is far greater than might be expected for a rare disease (*2*). Providing an online assessment reduced the need for in-person consultations from 242 to approximately 90, a 63% reduction. Because each human rabies death costs an estimated $191,000 in terms of staff member hours and PEP-associated costs, an online risk assessment could reduce administrative and financial costs (*10*). Since this evaluation, CDC has been improving tools available to states after mass rabies exposures and developing clearer content tailored for health care workers on human-to-human exposure risk in health care settings. Online tools that could be used in other events requiring numerous risk assessments appear to be an acceptable method to accurately assess exposure risk if they provide clear information on exposure and transmission pathways.

**Summary**

**What is already known about the topic?**

Human rabies cases are rare; however, exposure assessments to determine the need for postexposure prophylaxis (PEP) are time- and resource-consuming.

**What is added by this report?**

An online risk assessment tool was used following potential exposure to rabies virus in Utah. Among 90 health care workers identified by the tool as being potentially exposed to infectious material, 74 who were classified as exposed received PEP, after consultation with the occupational health staff, indicating a positive predictive value of 82%. In a follow-up survey, 42 (76%) of 55 participants reported satisfaction with the assessment process.

**What are the implications for public health practice?**

Online exposure assessment tools could substantially reduce the administration and financial obligation on health systems in events requiring numerous risk assessments; based on this evaluation, CDC is improving available tools for states in other mass rabies exposures.
